# Demographic and topographic findings suggesting poor response to crosslinking-iontophoresis in patients with progressive keratoconus

**DOI:** 10.1007/s10792-025-03421-9

**Published:** 2025-02-10

**Authors:** Rosario Touriño-Peralba, Julio Rodríguez-Lago, David Lamas-Francis, Laura Martínez-Pérez, Teresa Rodríguez-Ares

**Affiliations:** 1 Ocular Surface and Cornea Service, Department of Ophthalmology, University Hospital of Santiago de Compostela, Rúa Ramón Baltar s/n. PC: 15.706, Santiago de Compostela, A Coruna, Spain; 2 Department of Surgery, Faculty of Medicine, University of Santiago de Compostela, Santiago de Compostela, Spain

**Keywords:** Keratoconus, Cross-linking, Iontophoresis, Transepithelial, Tomography

## Abstract

**Purpose:**

To evaluate demographic and tomographical parameters in predicting treatment response following transepithelial iontophoresis-assisted corneal cross-linking (I-CXL) for progressive keratoconus.

**Methods:**

Forty eyes (20 aged < 19 years and 20 aged ≥ 19 years) underwent I-CXL treatment between 2016 and 2022. Progression criteria based on the ABCD system, changes in asphericity (Q), demographic factors and keratoconus phenotypes were evaluated. Subjects were followed for 24 months after procedure.

**Results:**

Sixty percent of participants were male. The mean age at the time of treatment was 21.0 ± 6.0 years. All tomographical values showed progression after 2 years of follow-up (*p* < 0.05), particularly during the first 6 months, except for anterior curvature. Within the ABCD grading system, we observed: A) an increase in anterior curvature, more evident with lower initial values; B) an increase in posterior curvature, more pronounced with higher initial values. Two years after I-CXL, 20% of subjects met progression criteria in two or more parameters, with 62.5% being under 19 years of age. Patients with a family history of corneal ectasia exhibited a mean KMax progression of 1.94D ± 1.88, (*p* = 0.046). Only phenotypes 3 and 4 showed progression. Although patients under 19 years showed greater progression in all tomographical variables at the end of the study, this difference was not statistically significant.

**Conclusion:**

Treatment with I-CXL did not stop progression in the variables studied two years after the procedure in an effective manner, especially in patients younger than 19 years. A family history of corneal ectasia and subtype 4 keratoconus predicted a less favourable response to I-CXL.

## Introduction

Keratoconus is an asymmetric ectatic disease, characterized by progressive thinning and steepening of the cornea, which lead to irregular astigmatism and decreased visual acuity [1, 2]. Keratoconus is a multifactorial condition influenced by genetics, environmental factors and inflammatory mediators [3].

Corneal tomography enables a detailed assessment of corneal features, including the curvature of the anterior surface, the elevation of both anterior and posterior corneal surfaces, corneal thickness and aberrometry. Modern tomography systems, such as the OCULUS Pentacam® have recently integrated advanced software applications like the Belin-Ambrosio Enhanced Ectasia Display for detecting subclinical keratoconus and the ABCD Belin Progression Display to monitor disease progression.

An expert panel [4] has defined the progression of keratoconus as an increase in anterior KMax, increased curvature of the anterior and/or posterior surfaces, thinning of the cornea and/or increase in the ratio between peripheral thickness and the thinnest point.

Management of keratoconus involves two complementary therapeutic strategies. Visual acuity can be improved with spectacles, contact lenses and intracorneal ring segments. When there is progression of keratoconus/corneal ectasia, the only technique that has been shown to stabilise the disease is corneal CXL. In some cases, corneal ablation procedures combined with cross-linking (CXL) to prevent disease progression and improve vision [5, 6]. The only technique shown to slow disease progression is corneal CXL. Keratoplasty is not considered as a treatment for preventing progression, but is used to enhance vision when other refractive techniques fail. However, recurrences of keratoconus in corneal transplant interfaces have been documented [7]. The primary components of CXL are a UV-A light source, a photosensitizer (riboflavin) and oxygen, which are crucial for maintaining the resulting photochemical reaction. The corneal epithelium acts as a barrier, limiting the transmission of UV light, riboflavin penetration and oxygen diffusion into the stroma. Although oxygen penetrates the epithelium and the endothelium, it does not diffuse effectively through the stroma [8]. Therefore, the corneal epithelium is removed in epithelium-off cross-linking (Epi-Off CXL). The Dresden protocol, based on this approach, has been proven to be effective in controlling keratoconus progression over a follow-up period exceeding 10 years [9, 10]. However, epithelial debridement is painful, delays recovery and increases the risk of infection [11]. Newer epithelium-on cross-linking (Epi-On CXL) techniques preserve the epithelium and use methods like iontophoresis, where an electrical field enhances riboflavin diffusion through the epithelium and stroma. Nonetheless, studies have shown that patients treated with iontophoresis have less favourable outcomes in reducing corneal curvature and slowing disease progression compared to standard Epi-off protocols [12].

The lack of a consensus on defining keratoconus progression complicates the interpretation of post-treatment results and hinders the ability to predict prognosis. This study aimed to evaluate the predictive ability of various tomographic parameters in assessing keratoconus progression in patients treated with transepithelial iontophoresis-assisted CXL (I-CXL) and to identify prognostic factors that can predict treatment response.

## Materials and methods

Forty patients with keratoconus treated with I-CXL at the University Hospital of Santiago de Compostela, Spain, between June 2016 and September 2022, were included.

All subjects met the keratoconus progression criteria established by the *Global Consensus on Keratoconus and Ectatic Diseases*, which considers changes in the anterior and posterior curvatures and the thinnest pachymetry [4]. However, as this definition lacks specific numerical values, additional criteria from other studies were also applied [13]. Progression was defined by the presence of at least two of the following tomographical changes in two consecutive medical visits separated by 6 months: a) an increase of > 1 diopter (D) in maximum keratometry (K-max), b) an increase of > 1 D in corneal astigmatism and c) a change in the posterior curvature of the cornea or d) a change of ≥ 5% in corneal pachymetry at the thinnest point.

All patients (n = 40) underwent a detailed ocular examination, with a particular focus on Distance Best Corrected Visual Acuity (DBCVA) with glasses and tomographic assessment, both before and after I-CXL treatment.

The tomographic parameters analysed using the ABCD Belin Progression Display included: anterior (A) and posterior curvature (B) in millimeters or diopters, both measured in the 3.0 mm zone centered on the thinnest point of the cornea; pachymetry (μm) at the thinnest location (C); and DBCVA (D), recorded on a decimal scale. Additional parameters included maximum keratometry (K-max) and corneal asphericity (Q).

The morphological and localisation patterns of keratoconus (phenotypes) were also assessed using the classification model proposed by Fernandez Vega et al., [14] which categorises keratoconus into: Type 1 or *croissant* (para- or pericentral), Type 2 or *duck* (paracentral), Type 3 or *snowman* (paracentral), Type 4 or *nipple* with (central) and Type 5 or *bow tie* (central).

All tomographical scans were performed using the Oculus Pentacam® (Oculus GmbH, Wetzlar, Germany). To enhance reproducibility, each patient underwent three scans at 5-min intervals.

Demographic data, tomographic parameters and morphological patterns of keratoconus according to the Fernández-Vega classification [14] are summarised in Table 1. DBCVA is displayed using the decimal scale. Tomographical parameters and DBCVA were evaluated before and after I-CXL (at 6, 12 and 24 months after the procedure). The follow-up period for all subjects was 2 years.

**Table 1:** List of demographic and tomographic features and morphological patterns (phenotypes) examined in this study

Demographic factors	Tomographic measurements	Morphological patterns*
Age at diagnosis	Maximum K	Croissant or type 1 (para or pericentral)
Age at time of I-CXL	Anterior and posterior corneal curvature	Duck or type 2 (paracentral)
Sex	Asphericity (Q)	Snowman or type 3 (paracentral)
Eye treated	Thinnest point pachymetry	Nipple or type 4 (central)
Previous ocular allergy / atopy		Bow-tie or type 5 (central)
Eye rubbing		
Family history of keratoconus		
Contact lens use		

^*^According to Fernández-Vega’s classification [14]

Exclusion criteria for this study included the presence of corneal ectatic diseases other than keratoconus, patients who did not meet progression criteria, those with a corneal thickness below 400 microns at the thinnest point, contraindications for I-CXL, prior intracorneal ring segment implantation or a follow-up duration of less than 24 months.

All participants provided informed consent prior to undergoing the procedure. For underage participants, consent was obtained from both the patient and a legal guardian. Ethical approval for the study was granted by the local ethics committee (2023/060), and the study adhered to the Code of Ethics of the World Medical Association (Declaration of Helsinki).

### Surgical technique: transepithelial I-CXL

The corneal iontophoresis system used was the only one marketed in Europe (Iontofor-CXL®), along with a specially formulated riboflavin solution designed for iontophoretic administration (Ricrolin + ®). This rivoflavin formulation rapidly penetrates the corneal stroma through the intact epithelium, allowing optimal impregnation without the need for epithelial removal.

The procedure began with the application of topical anesthetic eye drops (lidocaine 2%), followed by the disinfection of the periocular skin with alcohol to enhance electrode conductivity. The suction ring, containing an 8 mm-wide stainless-steel mesh (cathode), was positioned over the cornea (iontophor). The riboflavin solution (Ricrolin +) was used to fill the ring, covering the negative electrode, which remained submerged throughout the procedure. Both electrodes were connected to a power generator (I-ON CXL). An electrical current of 1 mA was applied for 5 min, followed by saline irrigation of the eye. UV-A light at 370 nm was then applied to the cornea at an intensity of 10 mW/cm^2^ for 9 min. During UV light exposure, preservative-free artificial tears were administered every minute. Post-procedure, patients were prescribed fluorometholone eye drops three times daily for two weeks, and diclofenac and cyclopentolate twice daily for the first two days. Preservative-free artificial tears with hyaluronic acid were prescribed the day after surgery every 2 h for the first two days, and then 4 times a day for 1 month. After surgery, no patient complained of significant discomfort, and conjunctival irritation was very mild in all patients.

### Statistical analysis

A descriptive analysis was performed. For qualitative variables, frequencies and percentages were calculated, while quantitative variables were expressed as mean and standard deviation, or median and range, depending on the data distribution. To compare groups and assess variables, Student’s t-test was used for comparisons between two groups, and ANOVA for multiple groups, provided that the data followed a normal distribution. If the data deviated from normal distribution, the Wilcoxon-Mann–Whitney test or the Kruskal–Wallis test was employed. A significance level of *p* < 0.05 was established. Statistical analyses were conducted using SPSS software version 23.0.

## Results

Forty eyes from 40 patients who underwent iontophoresis-assisted corneal cross-linking (I-CXL) were included in this study. None of the patients had received prior treatment for keratoconus. Among the participants, 20 patients were younger than 19 years, while 20 were 19 years or older. The treated eyes comprised 55% right eyes (22/40) and 45% left eyes (18/40).

### Demographic study

Sixty percent of the participants (24/40) were male, and 40% (16/40) were female. The mean age at the time of treatment was 21.0 ± 6.0 years (range: 13–37 years), and the mean age at diagnosis was 18.0 ± 5.0 years (range: 12–34 years). Allergies were reported in 22.5% of the patients (9/40), dermatitis in 25.0% (10/40), asthma in 10% (4/40), and rhinitis in 40% (16/40). Seventy-five percent (30/40) had a history of eye rubbing prior to their keratoconus diagnosis. Additionally, 17.5% had a family history of corneal ectasia (7/40), and 27.5% were contact lens wearers (11/40).

### Visual acuity (D-Belin Ambrosio Progression Display).

Distance Best Corrected Visual Acuity (DBCVA) was measured with glasses, using the decimal scale. The mean baseline DBCVA was 0.56 ± 0.23 (range: 0.10–1.00), while the mean DBCVA two years post-I-CXL was 0.64 ± 0.24 (range: 0.15–1.00). This improvement of 0.08 decimal points in DBCVA was statistically significant (*p* = 0.011).

### Tomographic study

The tomographic variables analyzed in this study are detailed in Table 2.

- 6 months post-treatment: Significant progression was observed in anterior curvature (*p* = 0.049), posterior curvature (*p* = 0.002), thinnest point pachymetry (*p* = 0.011), and K-max (*p* = 0.002).
- 12 months post-treatment: Significant progression was detected only in anterior curvature (*p* = 0.033) and thinnest point pachymetry (*p* < 0.001).
- 24 months post-treatment: All studied parameters except for anterior curvature showed significant progression: K-max (*p* = 0.005), asphericity (Q) (*p* = 0.014), posterior curvature (*p* = 0.022), and thinnest point pachymetry (*p* < 0.001).

**Table 2:** Analysis of tomographic variables studied before and after I-CXL treatment (at 6 months, 1 year and 2 years). Significant data are highlighted in bold and with asterisks

		∆KMax	∆Asphericity (Q)	∆Anterior curvature	∆Posterior curvature	∆Thinnest pachymetry
Mean ± S D	6 months	0.73 ± 1.38*	0.03 ± 0.22	0.45 ± 1.40*	0.15 ± 0.29*	− 7.86 ± (− 18.06)*
	1 year	0.34 ± 1.91	0.05 ± 0.22	0.38 ± 1.09*	0.07 ± 0.23	− 6.58 ± (− 10.83)*
	2 years	0.80 ± 1.70*	0.10 ± 0.26*	0.37 ± 1.28	0.08 ± 0.21*	− 8.75 ± (− 13.37)*
Median	6 months	0.50	0.03	0.20	0.10	− 3.00
	1 year	0.40	0.03	0.20	0.00	− 5.00
	2 years	0.70	0.04	0.25	0.10	− 6.50
Range	6 months	− 1.40 a 5.10	− 0.76 a 0.49	− 1.80 a 6.40	− 0.40 a 0.90	− 96.00 a 11.00
	1 year	− 6.20 a 4.50	− 0.67 a 0.58	− 1.70 a 4.50	− 0.40 a 0.70	− 44.00 a 14.00
	2 years	− 2.40 a 6.00	− 0.32 a 1.18	− 3.50 a 3.80	− 0.50 a 0.50	− 48.00 a 15.00
*p*-value	6 months	0.002*	0.434	0.049*	0.002*	0.011*
	1 year	0.263	0.185	0.033*	0.086	< 0.001*
	2 years	0.005*	0.014*	0.076	0.022*	< 0.001*

**∆:** change in the variables analyzed, SD: standard deviation

These are statistically significant results

**Fig. 1:**
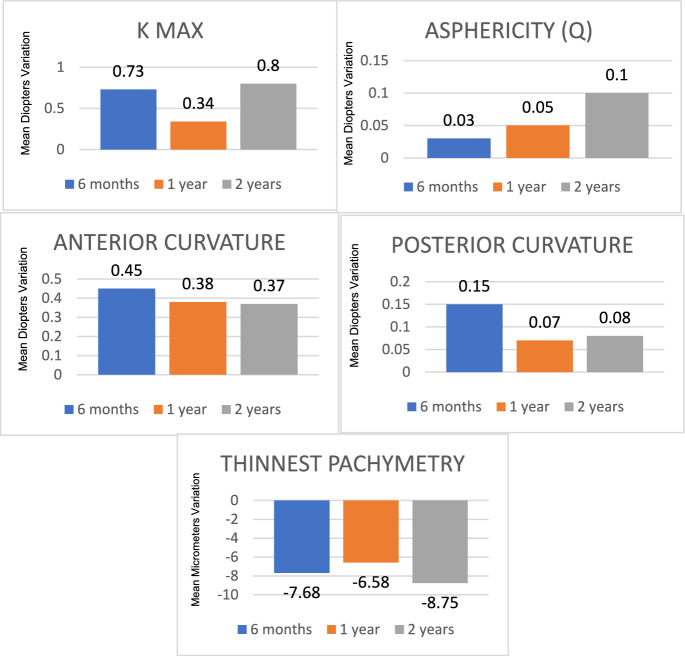
Mean progression rate of each tomographic variable at 6 months, 1 year and 2 years after I-CXL, compared to baseline values

We identified three key trends in the mean progression rate of each variable from baseline to 6, 12, and 24 months following I-CXL (Fig. 1).

Anterior and posterior curvatures showed their maximum difference from their baseline values at 6 months (+ 0.45D and + 0.15D, respectively) and regressed gradually until 24 months after treatment (+ 0.37D and + 0.08D from baseline).

- Anterior and posterior curvatures exhibited their greatest deviation from baseline at 6 months (+ 0.45D and + 0.15D, respectively), with a gradual regression observed up to 24 months post-treatment (+ 0.37D and + 0.08D from baseline).
- KMax and thinnest point pachymetry showed progression within the first 6 months (+ 0.75D and -7.86 µm, respectively), followed by regression between 6 and 12 months (+ 0.34D and -6.58 µm from baseline). However, these values increased again between 12 and 24 months post-treatment (+ 0.80D and -8.75 µm from baseline).
- Asphericity (Q) demonstrated a consistent progression throughout the study period, with mean increases of + 0.03D, + 0.05D, and + 0.10D at 6, 12, and 24 months, respectively.

When analyzing these same variables but grouping them into two age groups (< 19 years and ≥ 19 years), we observed the following trends (Table 3):

- **Under 19 years old**: Statistically significant progression was observed in Kmax at 6 months (0.73D ± 1.48), anterior curvature at 2 years (0.64D ± 1.18), posterior curvature at 6 months (0.24D ± 0.31), 1 year (0.14D ± 0.24), and 2 years (0.10D ± 0.20), and the thinnest pachymetry at 6 months (-11.70 µm ± 23.21), 1 year (-8.90 µm ± 11.71), and 2 years (-11.70 µm ± 12.52).
- **19 years or older**: Statistically significant progression was observed in Kmax at 6 months (0.72D ± 1.31), 1 year (0.75D ± 1.64), and 2 years (0.66D ± 1.27), as well as in corneal asphericity at 1 year (0.09D ± 0.17) and 2 years (0.10D ± 0.16).

**Table 3:** Analysis of tomographic variables studied before and after I-CXL treatment (at 6 months, 1 year and 2 years) dividing patients in those < 19 years and those ≥ 19 years

		∆KMax		∆Asphericity (Q)		∆Anterior curvature		∆Posterior curvature		∆Thinnest pachymetry	
		< 19 years	≥ 19 years	< 19 years	≥ 19 years	< 19 years	≥ 19 years	< 19 years	≥ 19 years	< 19 years	≥ 19 years
MEAN ± SD *P*-Value	6 months	**0.73 ± 1.48** *P* = 0.044*	**0.72 ± 1.31** *P* = 0.035*	0.04 ± 0.21 *P* = 0.397	0.01 ± 0.24 *P* = 0.191	0.65 ± 1.80 *P* = 0.204	0.25 ± 0.85 *P* = 0.126	**0.24 ± 0.31** *P* = 0.002*	0.06 ± 0.23 *P* = 0.215	− **11.70 ± 23.21** *P* = 0.007*	− 3.65 ± 9.83 *P* = 0.219
	1 year	− 0.06 ± 2.10 *P* = 0.481	**0.75 ± 1.64** *P* = 0.038*	0.00 ± 0.27 *P* = 0.872	**0.09 ± 0.17** *P* = 0.030*	0.55 ± 1.31 *P* = 0.144	0.21 ± 0.80 *P* = 0.162	**0.14 ± 0.24** *P* = 0.021*	− 0.01 ± 0.21 *P* = 0.874	− **8.90 ± 11.71** *P* = 0.001*	− 4.25 ± 9.60 *P* = 0.074
	2 years	0.94 ± 2.07 *P* = 0.064	**0.66 ± 1.27** *P* = 0.036*	0.10 ± 0.33 *P* = 0.179	**0.10 ± 0.16** *P* = 0.006*	**0.64 ± 1.18** *P* = 0.030*	0.10 ± 1.35 *P* = 0.643	**0.10 ± 0.20** *P* = 0.045*	0.06 ± 0.22 *P* = 0.178	− **11.70 ± 12.52** *P* < 0.001*	− 5.80 ± 13.85 *P* = 0.094

**∆**: change in the variables analyzed, SD: standard deviation

Significant data are highlighted with asterisks

These are statistically significant results

The analysis of patients who experienced significant progression in KMax (> 1D), anterior astigmatism (> 1D), or thinnest point pachymetry (> 5% from baseline) is detailed in Table 4. At 6 months, 7 patients showed progression in two or more variables, 5 patients showed progression within the first 12 months, and 8 patients exhibited progression at 24 months. This analysis was further stratified by age at the time of I-CXL (< 19 vs. ≥ 19 years) (Table 5).

**Table 4:** Number of patients showing progression of over 1D in KMax, > 1D in anterior astigmatism and > 5% in thinnest pachymetry, at 6, 12 and 24 months after treatment with I-CXL. Changes between 12 and 24 months are also provided

Variables analysed	0–6 months (%)	0—12 months (%)	12—24 months (%)	0—24 months (%)
KMax > 1D	14 / 40 (35%)	12 / 40 (25%)	11 / 40 (27.5%)	16 / 40 (40%)
Anterior astigmatism > 1D	9 / 40 (22,5%)	7 / 40 (17,5%)	3 / 40 (7,5%)	9 / 40 (22,5%)
Thinnest pachymetry (> 5%)	2 / 40 (5%)	3 / 40 (7,5%)	2 /40 (5%)	5 / 40 (12,5%)
KMax AND anterior astigmatism	6 / 40 (15%)	3 / 40 (7,5%)	2 / 40 (5%)	5 / 40 (12,5%)
KMax AND thinnest pachymetry (> 5%)	0 / 40 (0%)	1 / 40 (2,5%)	1 / 40 (2,5%)	1 / 40 (2,5%)
Anterior astigmatism AND thinnest pachymetry (> 5%)	1 / 40 (2,5%)	0 / 40 (0%)	0 / 40 (0%)	1 / 40 (2,5%)
KMax, AND anterior astigmatism AND thinnest pachymetry (> 5%)	0 / 40 (0%)	1 / 40 (2,5%)	0 / 40 (0%)	1 / 40 (2,5%)
Change in 2 or more variables	**7 (17,5%)**	**5 (12,5%)**	**3 (7,5%)**	**8 (20%)**

These are statistically significant results

**Table 5:** Number of patients showing progression of over 1D in KMax, > 1D in anterior astigmatism and > 5% in thinnest pachymetry, at 6, 12 and 24 months after treatment with I-CXL, according to age (< 19 years or ≥ 19 years)

Variables analysed	Under 19 years				≥ 19 years			
	0–6 months	0–12 months	12–24 months	0–24 months	0–6 months	0–12 months	12–24 months	0–24 months
KMax > 1D	6	5	8	11	8	6	3	5
Corneal astigmatism > 1D	6	5	1	5	3	3	2	4
Thinnest pachymetry (> 5%)	2	2	1	3	0	1	1	2
KMax AND corneal astigmatism	3	2	1	3	3	1	1	2
KMax AND Thinnest pachymetry (> 5%)	0	1	1	1	0	0	0	0
Corneal astigmatism AND Thinnest pachymetry (> 5%)	1	0	0	1	0	0	0	0
KMax AND corneal astigmatism AND Thinnest pachymetry (> 5%)	0	0	0	0	0	1	0	1
Change in 2 or more variables	**4 (10%)**	**3 (7,5%)**	**2 (5%)**	**5 (12,5%)**	**3 (7,5%)**	**2 (5%)**	**1 (2,5%)**	**3 (7,5%)**

These are statistically significant results

Among patients younger than 19 years, progression was observed in 4 cases within the first 6 months, 3 cases within the first year, and 5 cases during the first two years post-I-CXL. In contrast, for patients aged 19 years or older, progression was noted in 3 cases within the first 6 months, 2 cases within the first year, and 1 case within the first two years. At the 24-month follow-up, 20% of all subjects (8/40) showed progression in two or more tomographical variables, with 62.5% (5/8) of these cases occurring in patients younger than 19 years. When analyzed separately by age group, progression in those under 19 years was 12.5%, compared to a lower rate of 7.5% in those aged 19 years or older.

**Fig. 2:**
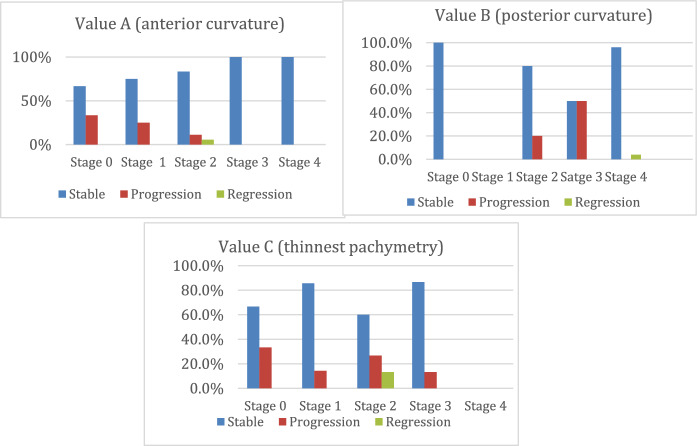
Evolution of Belin’s ABCD grading system, 2 years after treatment

Changes in the Belin ABCD Progression Display are illustrated in Fig. 2.

- **A-value** (anterior curvature of the cornea, averaged over the 3 mm zone around the thinnest point):
- At 2 years post-I-CXL, the percentage of patients remaining stable by category were: 66.7% in stage 0, 75% in stage 1, 83.3% in stage 2, and 100% in stages 3 or 4.
- Progression to higher stages occurred in 33.3% from stage 0 to 1, 25% from stage 1 to 2, 11.1% from stage 2 to 3.
- A regression was observed in 5.6% of patients from stage 2 to 1
- **B-value** (posterior curvature of the cornea, averaged over the 3 mm zone around the thinnest point):
- This value remained unchanged in 100% of cases at stage 0, 80% at stage 2, 50% at stage 3, and 96% at stage 4.
- Progression to higher stages was noted in 20% from stage 2 to 3 and 50% from stage 3 to 4.
- Only 4% of cases regressed from stage 4 to 3.
- **C-value** (thinnest point pachymetry):
- This value remained stable in 66.7% of patients at stage 0, 85.7% at stage 1, 60% at stage 2, and 86.7% at stage 3.
- Progression to higher stages occurred in 33.3% from stage 0 to 1, 14.3% from stage 1 to 2, 26.7% from stage 2 to 3, and 13.3% from stage 3 to 4.
- A regression was observed in 13.3% of patients from stage 2 to 1.
- **D-value** (Distance Corrected Visual Acuity, DCVA):
- Results for DCVA have been previously reported under Visual Acuity (D-Belin Ambrosio Progression Display).

### Combined analyses

- **Tomographic variables and demographic data** (Table 6):

- Eye rubbing: The mean progression of posterior curvature was 0.04 in patients who reported eye rubbing, compared to 0.18 in those who did not (*p* = 0.036).
- Allergies: A significant difference in asphericity (Q) was observed between allergic patients (mean progression -0.04) and those without allergies (0.16) (*p* = 0.005).
- Contact lens use: No significant difference was noted.
- Family history of corneal ectasia: KMax showed a mean progression of 1.94D in patients with a family history, compared to 0.56D in those without affected relatives (*p* = 0.046).
- Rhinitis: No significant difference was observed.
- Asthma: KMax showed a mean progression of -1.45D in asthmatic patients versus + 1.05D in non-asthmatic patients (*p* = 0.003).
- Age < 19 years: Although no statistically significant differences in progression were found between patients under 19 and those over 19, all studied variables showed a greater tendency towards progression in those younger than 19 years.

**Table 6:** Change in tomographical measurements after 2 years of treatment, compared to initial values, according to the presence of different demographic factors. Significant data are highlighted with asterisks

		∆KMax		∆Asphericity (Q)		∆Anterior curvature		∆Posterior curvature		∆Thinnest pachymetry	
		Mean	SD	Mean	SD	Mean	SD	Mean	SD	Mean	SD
Contact lens use	No	0,92	1,80	0,14	0,28	0,46	1,41	0,08	0,22	− 10,59	14,31
	Yes	0,49	1,45	0,00	0,14	0,14	0,87	0,06	0,16	− 3,91	9,39
Allergies	No	0,90	1,63	0,16*	0,25	0,21	1,25	0,09	0,22	− 8,35	14,66
	Yes	0,47	2,00	− 0,10*	0,17	0,90	1,29	0,03	0,12	− 10,11	7,94
Asthma	No	1,05*	1,59	0,12	0,26	0,36	1,30	0,08	0,20	− 9,03	13,28
	Yes	− 1,45*	0,77	− 0,01	0,19	0,48	1,15	0,08	0,29	− 6,25	16,05
Rhinitis	No	0,76	1,65	0,13	0,17	0,03	1,16	0,08	0,21	− 7,13	13,67
	Yes	0,86	1,83	0,07	0,35	0,87	1,32	0,07	0,20	− 11,19	12,94
Eye rubbing	No	1,23	1,31	0,07	0,19	0,87	1,19	0,18*	0,15	− 5,50	6,29
	Yes	0,66	1,81	0,12	0,28	0,20	1,28	0,04*	0,21	− 9,83	14,94
Family history of corneal ectasia	No	0,56*	1,59	0,09	0,27	0,34	1,33	0,07	0,22	− 9,06	13,63
	Yes	1,94*	1,88	0,16	0,21	0,50	1,04	0,11	0,13	− 7,29	12,94
Age < 19 years	No	0.66	1.27	0.10	0.28	0.10	1.35	0.06	0.22	− 5.80	13.85
	Yes	0.94	2.07	0.06	0.27	0.64	1.18	0.09	0.20	− 11.70	12.52

**∆** changes in the variables analyzed

These are statistically significant results

(b) **Tomographic Variables Based on Morphology and Localization of Keratoconus** (Table 7).

- **Phenotype 1** keratoconus was excluded from the analysis due to having only one case.
- Patients with **phenotypes 2 and 5** did not exhibit significant changes in any of the analyzed parameters.
- **Phenotype 3** keratoconus showed progression only in pachymetry (*p* = 0.024).
- **Phenotype 4** keratoconus demonstrated progression across all tomographic variables except for anterior curvature, with significant changes in KMax (*p* = 0.026), asphericity (Q) (*p* = 0.011), posterior curvature (*p* = 0.025), and thinnest point pachymetry (*p* = 0.006).

**Table 7:** Association between different types of keratoconus (Fernández-Vega classification)^14^ and the evolution of tomographic measurements, 2 years after treatment with I-CXL. Significant data are highlighted in bold and with asterisks. Subtype 1 was not included as only one case was examined

Type of keratoconus	∆KMax	∆Asphericity (Q)	∆Anterior curvature	∆Posterior curvature	∆Thinnest pachymetry
Type 2	0.753	0.236	0.799	0.084	0.553
Type 3	0.483	0.342	0.397	0.521	0.024*
Type 4	0.026*	0.011*	0.276	0.025*	0.006*
Type 5	0.173	0.249	0.116	0.336	0.058

**∆** changes in the variables analysed

These are statistically significant results

### Complications related to the technique

No patients developed complications after treatment or during the follow-up period.

## Discussion

Early detection is crucial in preventing the progression of keratoconus. While repeated examinations in individuals suspected of having keratoconus increase a clinic’s workload, they are essential for detecting progression and enabling early treatment to prevent further advancement of the disease. It is therefore important to identify which corneal parameters are involved in disease progression. Currently, no single variable can predict progression independently, so combinations of parameters are used: KMax, corneal astigmatism, pachymetry at the thinnest point, high-order comatic aberrations, visual acuity sphericity, and various risk factors. The Oculus Pentacam® was employed to evaluate our patients. Recently, the Oculus Pentacam® tomograph integrated the ABCD grading system, which appears to more accurately reflect the anatomical changes observed in keratoconus [15, 16]. This system includes anterior and posterior curvature measurements taken within a 3 mm zone centered around the thinnest point, the thinnest pachymetric values, and distance visual acuity, with grading from stage 0 to 4 (five stages in total). The aim of this study is to evaluate the changes in different tomographic variables (KMax, asphericity) and in the ABCD grading system in patients with progressive keratoconus treated with I-CXL. The response was also analyzed based on different keratoconus phenotypes (Fernández Vega classification) [14] and demographic factors.

The optimal timing for performing corneal cross-linking (CXL) is debated. Some authors [17] suggest performing CXL upon diagnosis, particularly in younger patients. However, complications such as infections or corneal haze can occur, potentially leading to decreased vision. Therefore, we believe that treatment should be based on documented disease progression. Conversely, older patients (> 30 years) are less likely to show progression and may derive less benefit from the procedure [18, 19]. In certain situations, immediate treatment may be justified, even without waiting for progression. This includes cases involving children with a strong tendency for ocular rubbing, poor patient or family compliance, or significant risk factors such as Down’s syndrome.

Risk factors for keratoconus progression recorded in this study included eye rubbing (75% of patients), allergic rhinitis (40%), ocular allergy (22.5%), atopic dermatitis (25%), asthma (10%), and a family history of corneal ectatic disease (17.5%). These results align with previously published findings by other researchers [20, 21]. Given these risk factors, it is crucial to educate the patient and family on the importance of stopping ocular rubbing. Additionally, accompanying ocular conditions should be treated with appropriate topical therapy, sometimes in collaboration with other specialists. This approach will alleviate the patient’s symptoms, reduce eye rubbing, and decrease ocular surface inflammation, which may contribute to the progression of keratoconus.

The mean DBCVA improved by 0.08 points (*p* = 0.011) in our study, consistent with other reports showing improvements of 0.14 or 0.17 decimal points [22, 23]. The primary aim of CXL is not to improve vision but to prevent further vision loss. Other treatments, such as contact lenses, intracorneal ring segments, and corneal ablation procedures combined with CXL, can improve vision in certain cases [5, 6]. When other refractive techniques fail, keratoplasty is a viable option.

The most common keratoconus phenotype in our series was phenotype 4 (nipple), observed in 37.5% of cases, followed by phenotype 3 (snowman) in 22.5%. The only phenotypes that showed progression were type 3 (thinnest pachymetry) and type 4 (all variables except anterior curvature). Although type 3 has a paracentral morphology, type 4 is entirely central, suggesting that CXL should be more effective by targeting the central 8 mm. Phenotype 4 is more prevalent in younger patients, in whom keratoconus tends to progress more aggressively [19]. These findings, not previously reported in other studies, suggest a need for more vigilant monitoring and potentially more aggressive treatment, such as removing the corneal epithelial layer (traditional CXL following the Dresden protocol or accelerated Epi-Off CXL techniques).

We observed significant differences in all tomographical values except anterior curvature two years post-I-CXL. These findings differ from other studies that only evaluated KMax and pachymetry, reporting improvements in KMax of -2.30 ± 5.01D (*p* = 0.014) [22] and no difference in the thinnest pachymetry [24].

Tomographic variables, except for asphericity, showed notable progression in the first 6 months following I-CXL. Some authors have reported progression changes within weeks of undergoing treatment with Epi-Off CXL [25, 26]. Other studies have reported similar changes in the in pachymetry post-I-CXL, with significant thinning in the initial months that returns to baseline over time [24, 27]. This initial decrease in pachymetry should not be interpreted as disease progression but rather as a compression of stromal collagen fibers due to CXL [28, 29].

The extent of corneal thinning after CXL could indicate the intensity of this cross-linking effect. Other parameters may initially change due to the treatment itself rather than due to disease progression. Considering only the first 6 months post-I-CXL, 7 patients would meet the progression criteria, while 5 would be classified as progressing at 1 year and 8 at 2 years. However, if only the period between 1 and 2 years is considered, only 3 patients would meet the criteria, indicating the need for further treatment. Differentiating between progression and changes induced by CXL is crucial for accurate assessment. Based on these findings, we believe that caution is necessary when assessing possible progression or lack of response to treatment, as it may be a "pseudoprogression."

Belin’s ABCD grading system allows for a rapid evaluation of each variable. Subjects with a lower baseline A score (anterior curvature) showed a stronger tendency towards progression than those with higher baseline scores, whereas the opposite trend was observed with B scores (posterior curvature). The C score did not follow this pattern. These findings suggest that keratoconus treated with I-CXL is more likely to progress in cases with lower A scores (0 or 1) and higher B scores (3 or 4), despite treatment.

Many studies have investigated risk factors associated with the development and progression of keratoconus prior to treatment, but few have analyzed progression following CXL. Eye rubbing [30], a KMax > 58.0D [31], and age under 19 years [17] have been proposed as risk factors for progression despite treatment. We observed significantly greater progression in KMax among patients with a family history of ectatic disease, highlighting the need for vigilant monitoring in this subgroup. However, some results were less straightforward, such as a higher progression rate in posterior curvature among patients who do not rub their eyes, decreased asphericity in allergic patients, and increased KMax in asthmatic patients but decreased KMax in non-asthmatic patients. These findings may be due to undetected biases, the small study population, or the fact that patients who rub their eyes are advised to stop, while those who do not are not given similar instructions.

Some researchers consider I-CXL to be less effective in younger patients [17, 32] than classical CXL (Dresden protocol) or accelerated Epi-Off CXL [12, 32, 33]. In our study, there was progression in more topographic variables (thinnest pachymetry, anterior curvature, and posterior curvature) in patients under 19 years of age, whereas those aged 19 years or older showed progression only in Kmax and asphericity. Furthermore, the group under 19 years demonstrated progression in at least two variables across all examined time periods, indicating a reduced therapeutic effect in younger patients. However, this could not be confirmed due to a lack of statistical significance, warranting further studies. Alternative Epi-Off techniques could potentially be more effective in this age group.

This study presents findings following I-CXL for progressive keratoconus over a longer period than other studies. To our knowledge, it is the only study to examine the morphology of keratoconus and its response to treatment. However, this study has several limitations, including its retrospective design and small sample size. Longer-term studies are needed to confirm the effectiveness of this technique.

In conclusion, the use of I-CXL for progressive keratoconus in patients under 19 years of age showed greater progression in all variables analyzed two years post-procedure, although this was not statistically significant. A family history of corneal ectasia and subtype 4 keratoconus were associated with a less favorable response to I-CXL.

## Data Availability

No datasets were generated or analysed during the current study.
